# Diagnostic Value of Gastrointestinal Ultrasound (GIUS), Including Doppler and Contrast-Enhanced Ultrasound (CEUS), in the Assessment of Inflammatory Bowel Disease Activity: A Scoping Review

**DOI:** 10.3390/jcm15207984

**Published:** 2026-10-15

**Authors:** Radosław Korczyk, Angelika Złotnik, Paweł Ścierski, Barbara Lipka, Piotr Oleś, Aleksandra Kawczyk-Krupka

**Affiliations:** 1 Department of Internal Medicine, Angiology and Physical Medicine, Medical University of Silesia in Katowice, 40-055 Katowice, Poland; 2 Faculty of Medical Sciences in Zabrze, Medical University of Silesia in Katowice, 40-751 Katowice, Poland

**Keywords:** gastrointestinal ultrasound, inflammatory bowel disease, contrast-enhanced ultrasound, Doppler ultrasound, Crohn’s disease, ulcerative colitis, bowel wall thickness

## Abstract

**Background/Objectives:** Treat-to-target management of inflammatory bowel disease (IBD) requires frequent, accurate assessment of disease activity. Endoscopy remains the reference standard, but its invasiveness limits repeated use, which has increased interest in gastrointestinal ultrasound (GIUS). To map the evidence on the diagnostic value of GIUS, including Doppler and contrast-enhanced ultrasound (CEUS), for IBD activity, with reference to endoscopy and serological/immunological biomarkers. **Methods:** This scoping review followed PRISMA-ScR. PubMed, Scopus, Web of Science and Embase were searched from January 2018 to March 2026, supplemented by reference-list screening. Two reviewers screened records independently. No formal risk-of-bias assessment was performed, and the synthesis was narrative. **Results:** Of 1284 records, 15 primary studies were included. Published meta-analyses report sensitivity of 85–91% and specificity of 83–96% for GIUS versus endoscopy; these are ranges from published sources, not estimates pooled by us. Bowel wall thickness (BWT) > 3 mm was the most frequently evaluated parameter. Color Doppler (Limberg grading) and CEUS added vascularity and perfusion information, and CEUS showed the highest reported accuracy in the meta-analytic literature, although data are limited. Combined GIUS–biomarker approaches reported accuracies of about 88–92% in single-center studies. Cytokine data (including IL-8) came mainly from non-GIUS studies and are hypothesis-generating. **Conclusions:** GIUS, alone or with biomarkers, is a promising complementary tool for monitoring IBD, mainly in moderate-to-severe disease. Because the evidence is heterogeneous, of unassessed quality and lacks randomized comparisons, it does not currently support replacing endoscopy or deferring it safely. Standardized protocols and randomized trials are needed.

## 1. Introduction

Inflammatory bowel disease (IBD), comprising Crohn’s disease (CD) and ulcerative colitis (UC), requires frequent and accurate assessment of disease activity to guide therapeutic decisions and optimize patient outcomes. Endoscopy has traditionally served as the reference standard for determining mucosal inflammation and monitoring treatment response [1,2]. However, the invasive nature of colonoscopy and ileocolonoscopy, together with patient discomfort, procedural risk and resource constraints, limits their feasibility for repeated monitoring in clinical practice [3,4]. The evolution toward treat-to-target strategies has intensified the need for reliable, non-invasive methods of assessing disease activity [2]. Gastrointestinal ultrasound (GIUS), comprising conventional B-mode imaging, Color Doppler imaging (CDI) and contrast-enhanced ultrasound (CEUS), is a point-of-care modality that offers real-time assessment without radiation exposure or extensive patient preparation [3,5]. Published meta-analyses report sensitivity of about 85–91% and specificity of 83–96% for GIUS against endoscopy in detecting active inflammation [4,6,7]; these are ranges reported by others, not estimates pooled in this review.

### 1.1. Ultrasound Parameters and Disease Activity

**Figure 1:**
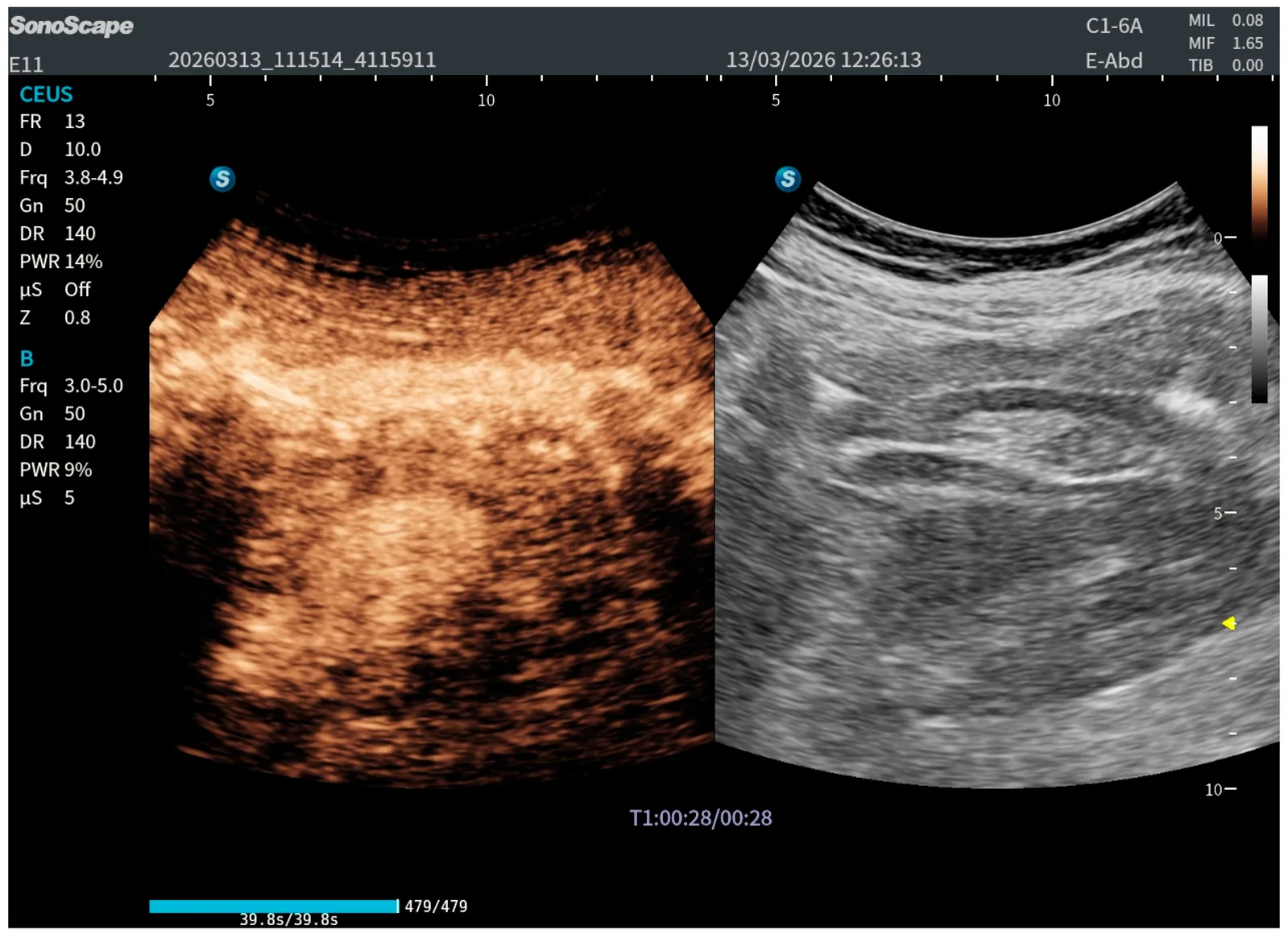
CEUS of the colon in a patient with suspected IBD flare (dual-panel display: contrast-enhancement map on the **left** and B-mode image on the **right**). Later post-contrast phase (approx. 39.8 s) shows persistent hyperenhancement predominantly within the mucosal layer, supporting localization of active inflammation to the colonic mucosa.

**Figure 2:**
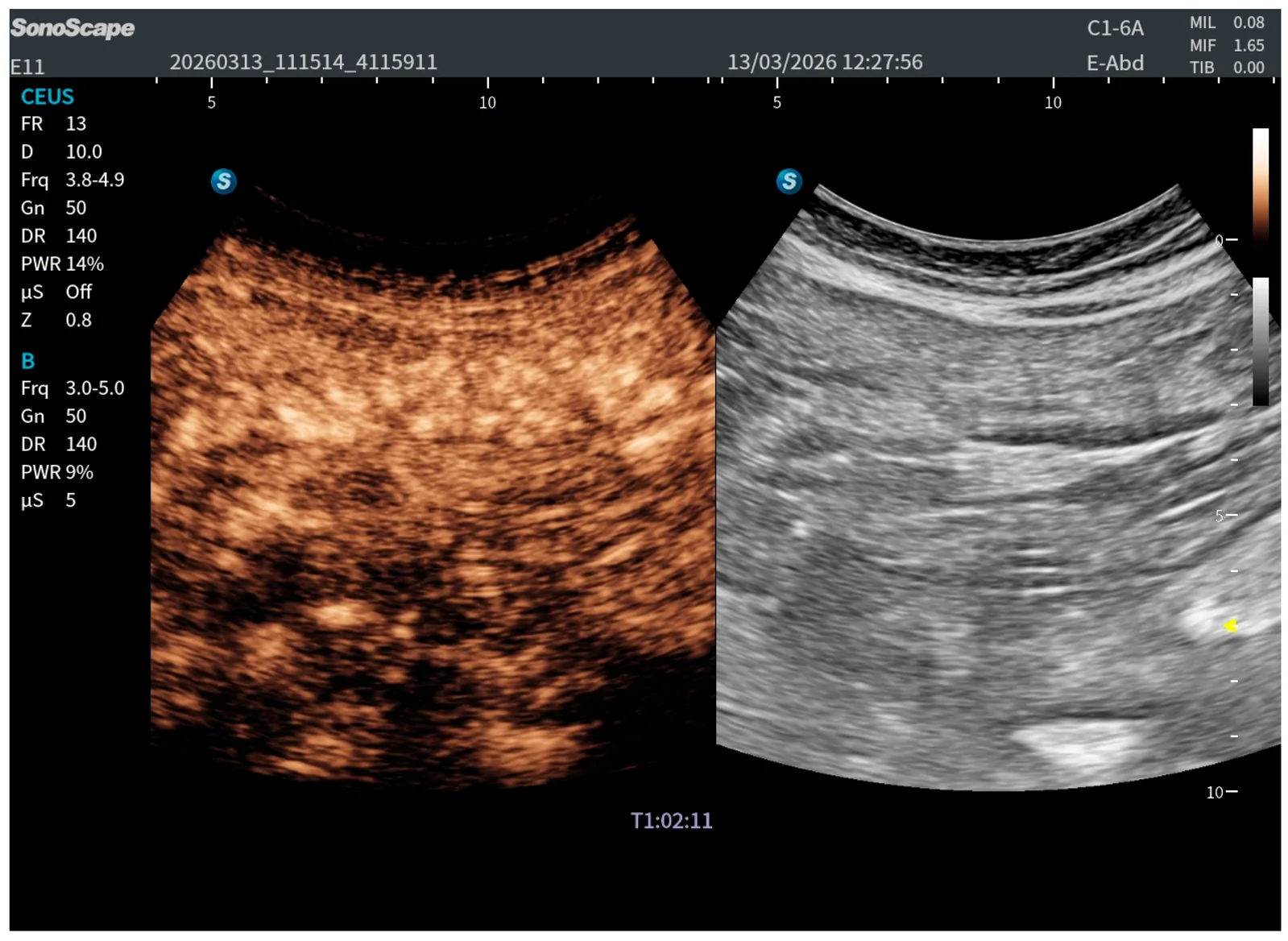
CEUS of the colon in a patient with suspected IBD flare (dual-panel display: contrast-enhancement map on the **left** and B-mode image on the **right**). Delayed post-contrast image demonstrates that mural enhancement persists for >120 s after microbubble injection (time stamp at the bottom of the image). Persistent enhancement reflects ongoing hyperperfusion of the inflamed bowel wall, consistent with sustained microvascular recruitment (dilated vasa recta, angiogenesis) and increased regional blood volume typical of active inflammation. As microbubble contrast agents remain intravascular, the signal intensity in the bowel wall primarily reflects microvascular perfusion rather than extravascular leakage.

Several sonographic parameters are used as markers of disease activity. Bowel wall thickness (BWT) greater than 3 mm is the most widely used and correlates with endoscopic severity [6,8]. Color Doppler assessment with the Limberg scale (grades 0–3; some studies use a modified 0–4 version) quantifies intestinal wall vascularity, with higher grades corresponding to more active inflammation [9,10]. CEUS provides quantitative perfusion parameters, including time to peak enhancement (TTP), peak intensity (PI) and the area under the time–intensity curve, and has shown accuracy comparable to or exceeding conventional ultrasound [4,11]. Foundational studies published before 2018, which lie outside the review window and are cited as background only, linked CEUS enhancement parameters and Doppler vascularity to endoscopic and surgical findings [12,13,14]. An example CEUS screen is shown in Figure 1 and Figure 2.

### 1.2. Biomarkers in IBD Assessment

C-reactive protein (CRP) has reasonable sensitivity but limited specificity (about 85% and 61%, respectively) [7]. Fecal calprotectin performs better for endoscopic remission in UC (AUC 0.885–0.891) but discriminates poorly between moderate and severe activity [15,16]. Cytokines are an emerging area. A pre-2018 study (background only) using the Bio-Plex multiplex system found that UC patients had higher IL-8 (OR 1.37, *p* = 0.002) and IL-10 (OR 3.88, *p* = 0.012) and lower IFN-γ (OR 0.95, *p* = 0.002) than controls, with a diagnostic accuracy of 77.3% overall and 94.6% in newly diagnosed patients; IL-8 correlated with endoscopic (OR 1.10, *p* = 0.026) and histological severity (OR 1.33, *p* = 0.017) [17]. A four-marker serum panel (SAA, IL-6, IL-8, Eotaxin-1) predicted endoscopic activity with an optimism-adjusted AUC of 0.84 (95% CI 0.73–0.94), compared with 0.57 for CRP alone [18]. Mucosal IL-8 expression has been linked to later relapse in UC with a Mayo endoscopic subscore of 0 [19]. Other multiplex cytokines (including IL-5, IL-8, IL-9, IL-10, IL-12, PDGF-BB and RANTES) have been correlated with disease activity indices, MRI findings and fecal calprotectin [20], and IL-6, IL-1β and IL-10 with disease activity and nutritional status [21]. None of these studies assessed GIUS, so they are presented as biomarker background.

### 1.3. Integration of GIUS and Biomarkers

Combining GIUS parameters with inflammatory biomarkers is an attractive strategy that might allow accurate monitoring with less frequent endoscopy, particularly in established disease in clinical remission [3,5]. Recent guidelines from the American College of Gastroenterology (2025) and the American Gastroenterological Association (2024) recognize intestinal ultrasound as a valuable tool for measuring disease activity and monitoring treatment response, with response detectable as early as 2 weeks after starting therapy [1,3]. However, the AGA biomarker guidelines find the evidence insufficient to recommend biomarker-based monitoring as a replacement for endoscopic assessment [15,22]. They stress that fecal calprotectin and CRP can help rule out active inflammation in symptomatic remission, but that elevated biomarkers do not always indicate active disease and normal values cannot exclude inflammation in patients with mild symptoms [15,22].

### 1.4. Rationale and Objectives

Important questions remain. The optimal combination of ultrasound parameters and biomarkers has not been systematically defined across populations and clinical scenarios. The comparative performance of B-mode, Doppler and CEUS needs clarification, including cost-effectiveness and practical implementation [4,23]. The role of advanced inflammatory markers, particularly cytokines measured on multiplex platforms such as the Bio-Plex Pro™ Human Inflammation Panel, needs evaluation to determine their incremental value over established biomarkers [17,18,20].

This scoping review aims to map current evidence on the diagnostic value of GIUS, including Doppler and CEUS, in assessing IBD activity with reference to endoscopic findings and serological/immunological biomarkers.

## 2. Methods

### 2.1. Design and Reporting

This scoping review evaluates the diagnostic value of GIUS, including Doppler ultrasound and CEUS, in assessing IBD activity with reference to endoscopy and biomarkers. A systematic review with meta-analysis was not feasible because of substantial heterogeneity, so the aim was to map current knowledge and to inform future studies with our team. The review is reported according to the PRISMA Extension for Scoping Reviews (PRISMA-ScR) [24]; the completed checklist is provided as Supplementary Material. A meta-analysis was not planned or performed. A protocol defining the question, eligibility criteria, search strategy, selection process and data extraction was agreed internally before the search but was not prospectively registered.

The question was structured with PICO elements:

- Population: Patients with IBD (Crohn’s disease or ulcerative colitis).
- Index test: GIUS, including B-mode ultrasound, Doppler ultrasound and CEUS.
- Comparator: Endoscopic assessment (colonoscopy or ileocolonoscopy) and/or inflammatory biomarkers (CRP, fecal calprotectin, cytokines such as IL-8, IL-6, IL-10, IFN-γ).
- Outcome: Diagnostic accuracy and correlation with disease activity (sensitivity, specificity, AUC, correlation coefficients).

The primary objective was to map the diagnostic accuracy of GIUS parameters in detecting active IBD compared with endoscopy. Secondary objectives were to map correlations between ultrasound findings and biomarkers and the performance of combined approaches.

### 2.2. Search Strategy

PubMed, Scopus, Web of Science and Embase were searched for publications from January 2018 to March 2026 (last search: March 2026), combining Medical Subject Headings (MeSH) and free-text terms for IBD, ultrasound modalities and biomarkers. The core query was: (“inflammatory bowel disease” OR “Crohn’s disease” OR “ulcerative colitis”) AND (“intestinal ultrasound” OR “gastrointestinal ultrasound” OR “GIUS” OR “bowel ultrasound” OR “contrast-enhanced ultrasound” OR “CEUS” OR “Doppler ultrasound”) AND (“disease activity” OR “endoscopy” OR “endoscopic activity”) AND (“biomarkers” OR “C-reactive protein” OR “fecal calprotectin” OR “cytokines” OR “IL-8” OR “IL-6” OR “IL-10”). Database-specific formulations are given in Appendix A. Reference lists of eligible studies and relevant reviews were also screened manually. The mandatory biomarker block may have reduced sensitivity for studies comparing GIUS with endoscopy only (see Section 4.6).

### 2.3. Eligibility Criteria

Inclusion criteria:

1. Original clinical studies (any design) evaluating GIUS, Doppler ultrasound or CEUS in patients with Crohn’s disease or ulcerative colitis.
2. Studies assessing disease activity using ultrasound parameters such as bowel wall thickness, vascularity or contrast perfusion metrics.
3. Studies comparing ultrasound with endoscopic findings, histology or inflammatory biomarkers.
4. Studies reporting diagnostic performance measures (sensitivity, specificity, correlation coefficients or AUC).
5. Studies published between January 2018 and March 2026 in peer-reviewed English-language journals.

Exclusion criteria:

- Case reports or case series with fewer than 10 patients.
- Animal or in vitro studies.
- Narrative reviews, editorials and conference abstracts without full data.
- Studies lacking objective assessment of disease activity.
- Studies not reporting ultrasound parameters relevant to disease activity.

Systematic reviews, meta-analyses and guidelines were not counted among the included studies. They were used only as sources of pooled estimates (attributed explicitly) and of clinical context. Publications before 2018 are cited as background only and are marked as such.

### 2.4. Data Charting

Data were charted with a standardized form: study characteristics (authors, year, country, design); population (IBD subtype, sample size, disease phenotype); ultrasound modality (B-mode, Doppler, CEUS); sonographic parameters (BWT, Limberg score, CEUS perfusion metrics); comparators (endoscopic scores, histology, biomarkers); biomarkers evaluated (CRP, fecal calprotectin, cytokines such as IL-8, IL-6, IL-10); and diagnostic performance metrics (sensitivity, specificity, AUC, correlation coefficients).

### 2.5. Quality Appraisal and Synthesis

Consistent with scoping-review methodology, no formal risk-of-bias assessment was performed; conclusions are therefore drawn cautiously. Because of heterogeneity in ultrasound protocols, scoring systems, biomarker panels and outcome definitions, findings were synthesized narratively, focusing on: (1) diagnostic accuracy of individual GIUS parameters; (2) correlation between ultrasound findings and endoscopic scores; (3) associations between GIUS parameters and inflammatory biomarkers; and (4) performance of combined GIUS–biomarker approaches. Performance metrics are reported as ranges from individual studies, or as pooled estimates only when taken from a published meta-analysis, which is then cited.

## 3. Results

### 3.1. Study Selection

**Figure 3:**
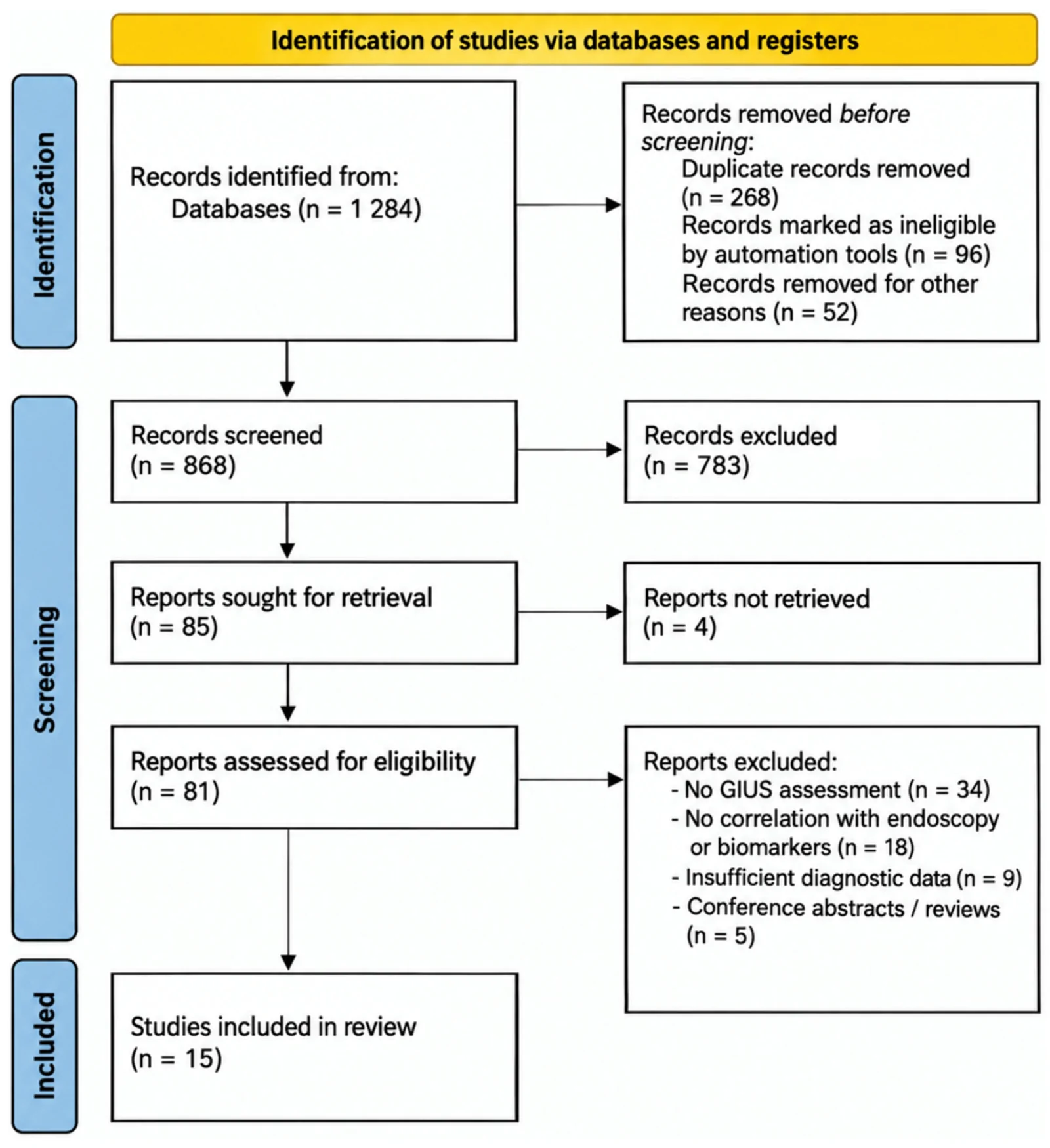
PRISMA-ScR flow diagram of study selection.

The literature search initially identified 1284 records from databases. After removing 268 duplicate records, 96 records marked as ineligible by automation tools, and 52 records removed for other reasons, 868 records remained for screening. Of these, 783 records were excluded, leaving 85 reports sought for retrieval. Four reports could not be retrieved, resulting in 81 reports assessed for eligibility. During the eligibility assessment, 34 reports were excluded because they did not include a GIUS assessment, 18 lacked correlation with endoscopy or biomarkers, 9 had insufficient diagnostic data, and 5 were conference abstracts or reviews. Ultimately, 15 studies were included in the systematic review (Figure 3). 

### 3.2. Principal Findings

Published meta-analyses report GIUS sensitivity of 85–91% and specificity of 83–96% against endoscopy [4,6,7]. For comparison, CRP showed similar sensitivity (about 85%) but lower specificity (about 61%) [7]; these comparisons are indirect, made across studies. Studies combining GIUS parameters with biomarkers reported accuracies of about 88–92% for endoscopic and histological activity [25,26,27], although these were mostly small, single-center studies.

### 3.3. Bowel Wall Thickness

BWT was the most frequently evaluated parameter. A meta-analysis of colorectal segments reported pooled sensitivity of 86.4% and specificity of 88.3% for BWT > 3 mm [6]. Performance was lower in the rectum (sensitivity 74.5%, specificity 69.5%) [6]. In a prospective UC cohort receiving tofacitinib, BWT correlated with the endoscopic Mayo score (ρ = 0.68, *p* < 0.0001); the optimal cut-offs were 2.8 mm (AUC 0.87) for endoscopic remission and 3.9 mm (AUC 0.92) for endoscopic improvement [8]. These AUCs apply to that single study and are not a general range for BWT > 3 mm. In some studies BWT correlated better with endoscopic activity than fecal calprotectin, and BWT changes were detectable as early as 2 weeks after starting therapy [8,28,29].

### 3.4. Color Doppler

Several studies report positive correlations (ρ about 0.70–0.90) between Limberg grading and endoscopic activity indices [9,10,30]. In a CD study of 125 patients, Limberg grade correlated with the Simple Endoscopic Score for CD (SES-CD; ρ = 0.709, *p* < 0.001), and cases with Limberg grade 3 or 4 were endoscopically active (modified scale) [30]. Earlier work before the search window (background only) also found Limberg grade to correlate with surgical macroscopic activity, supporting its relevance for transmural inflammation [13]. Limberg scales differ between studies (0–3 versus modified 0–4), which limits comparability.

### 3.5. Contrast-Enhanced Ultrasound

CEUS adds perfusion parameters, of which TTP and PI showed the strongest associations with disease severity [9,11]. Against surgical histopathology, TTP and rise time reached AUCs of 0.88 and 0.86 for severely active CD [11]. A meta-analytic estimate attributes the highest diagnostic odds ratio (75.52) to CEUS, with sensitivity of 93.5% and specificity of 93.7% for inflammatory activity [4]. A pre-2018 study (background only) proposed peak-enhancement cut-offs of 18.2 dB for mild versus moderate activity (sensitivity 89.0%, specificity 87.0%) and 23.0 dB for moderate versus severe activity (sensitivity 90%, specificity 86.8%) [14]. A multicenter score combining BWT, Limberg grade and CEUS parameters reached an AUC of 0.972 for active CD, and 0.923 without the CEUS parameters [9]. These estimates rest on few, heterogeneous studies, so CEUS superiority remains preliminary.

### 3.6. Composite Ultrasound Scores

Several scores have been proposed, including IBUS-SAS, BUSS, Simple-US and SUS-CD. In one prospective study IBUS-SAS showed the highest correlation with endoscopy (ρ = 0.87) but requires more granular assessment [31]. A score combining BWT, Limberg grade, disease extension and elastography discriminated remission from relapse at a cut-off of 7 points (sensitivity 86.85%, specificity 94%) [10]. Multiparametric scores seem to capture disease activity more comprehensively than single parameters, and validation studies of the available scores are ongoing [23].

### 3.7. Combined GIUS–Biomarker Approaches

In 45 patients with CD, BWT > 3 mm plus fecal calprotectin > 250 µg/g gave sensitivity/specificity of 88.6%/78.9% in the ileum and 80.9%/87.7% in the descending colon [25]. In UC, composite assessment with intestinal ultrasound and calprotectin predicted histological activity with an accuracy of 88% (*p* = 0.007), sensitivity of 88%, specificity 80%, PPV 95% and NPV 57% [26]. The low NPV limits its use for ruling out activity. In pediatric IBD, adding intestinal ultrasound to clinical parameters improved prediction of biochemical activity in CD (AUC 0.71 versus 0.90; *p* = 0.004) and, non-significantly, in UC (AUC 0.83 versus 0.92; *p* = 0.067) [27]. Optimal BWT thresholds for predicting fecal calprotectin > 250 µg/g were 2.3–2.5 mm depending on phenotype, and median BWT was 4.2 mm in active versus 2.0 mm in quiescent disease (*p* < 0.001) [27].

### 3.8. Comparison with MR Enterography and Endoscopy

GIUS has shown accuracy comparable to MR enterography (MRE) for terminal ileal activity, with practical advantages: point-of-care availability, no radiation and no oral or intravenous contrast [3,32]. In a multicenter trial summarized in the AGA commentary, intestinal ultrasound and MRE had similar accuracy in newly diagnosed or clinically active CD, with higher interobserver agreement for ultrasound (82%, κ = 0.64) than for MRE (69%, κ = 0.36) [3]. For small-bowel involvement, GIUS sensitivity of about 92% has been described against 97% for MRE [32]. Endoscopy retains distinct advantages: direct mucosal visualization and tissue sampling for histology. GIUS performs best in moderate-to-severe activity, when wall thickening > 3 mm, submucosal edema and hyperperfusion are present, and less well in endoscopically mild disease and in the rectum [3,6].

### 3.9. Prediction of Disease Course and Treatment Response

In a 12-month prospective study of 225 patients with CD, a bowel ultrasound score > 3.52 independently predicted adverse outcomes (steroids, therapy change, hospitalization or surgery; OR 6.97, 95% CI 2.87–16.93, *p* < 0.001), similar to fecal calprotectin ≥ 250 µg/g (OR 5.43, 95% CI 2.25–13.11, *p* < 0.001); combining the two improved risk stratification [33].

A systematic review in UC summarized early dynamic changes as predictors of outcome [29]. In hospitalized patients receiving intravenous corticosteroids, BWT ≥ 4 mm at 48 h (AUC 0.77), an absolute reduction ≤ 1 mm (AUC 0.71, 95% CI 0.56–0.86) or a relative reduction ≤ 20% (AUC 0.74, 95% CI 0.60–0.88) identified patients needing rescue therapy; BWT < 3 mm at 48 h was associated with no colectomies, whereas BWT ≥ 4 mm or persistently raised BWT at day 6 increased colectomy risk (OR 9.5, 95% CI 1.4–64.0; and OR 8.3, 95% CI 1.7–40.0) [29]. In outpatients, BWT ≤ 3.6 mm at 2 weeks and <3.0 mm at 6 weeks were associated with early endoscopic remission (AUROC 0.87, 95% CI 0.71–1.00; and 0.82, 95% CI 0.63–1.00), and a relative reduction of ≥23–25% predicted clinical or endoscopic response (AUROC 0.81, 95% CI 0.61–1.00; OR 13.9, 95% CI 1.13–1986.85, an imprecise estimate) [29]. Milan Ultrasound Criteria (MUC) ≤ 4.3, or a ≥2-point reduction at 12 weeks, predicted long-term remission (AUROC 0.88, 95% CI 0.750–0.952; and 0.82, 95% CI 0.68–0.91), while MUC ≥ 7.7 indicated high risk of treatment failure or colectomy (AUROC 0.77, 95% CI 0.73–0.82) [29]. These estimates come from small studies.

### 3.10. Postoperative Monitoring, Point-of-Care Use and Trial Pre-Screening

In postoperative CD, BWT ≥ 3 mm in the neo-terminal ileum or adjacent colon combined with fecal calprotectin ≥ 50 µg/g detected recurrence with PPV 74% (95% CI 63–84%) and NPV 71% (95% CI 42–92%) [34]. Pooled sensitivity was 0.76 (95% CI 0.70–0.82) for fecal calprotectin at the 50 µg/g threshold and 0.92 (95% CI 0.75–0.96) for cross-sectional imaging [34]. These figures suggest such tools can reduce, but not yet replace, colonoscopic monitoring. GIUS combined with point-of-care or home biomarker testing allows treatment adjustment during a clinic visit without the delay of endoscopy scheduling [35,36]. In pregnancy, where avoiding radiation and sedation matters, intestinal ultrasound showed moderate-to-strong correlation with clinical activity and fecal calprotectin (sensitivity 84%, specificity 98%), with the colon and terminal ileum visualized in up to 92% of third-trimester examinations [3]. For clinical-trial pre-screening, segmental BWT thresholds (≤3.1 mm for SES-CD ≤ 2; ≥3.6 mm for SES-CD ≥ 6) identified endoscopic severity with AUCs of 0.833–0.950 [37].

### 3.11. Cytokine Biomarkers

The cytokine evidence in Section 1.2 comes from studies without GIUS. IL-8 correlated with disease severity in these studies and mucosal IL-8 with later relapse [17,19], and multi-cytokine panels outperformed CRP alone [18]. A correlation does not, however, establish IL-8 as the most reliable or clinically superior biomarker, and no GIUS study mapped here demonstrated incremental value of IL-8 over calprotectin. Erythrocyte sedimentation rate was rarely the focus of the identified studies, most of which examined CRP and fecal calprotectin. A summary of this information is available in Table 1 and Table 2.

## 4. Discussion

### 4.1. Key Findings

The mapped evidence suggests that GIUS can detect endoscopically active IBD with useful accuracy, particularly through BWT in moderate-to-severe disease and in colonic segments. Doppler and CEUS add vascular and perfusion information, but the CEUS evidence base is small and its advantage is not firmly established. Combinations with calprotectin reached accuracies of about 88–92% in small, single-center studies, but their NPVs (57–71% in the studies above) are too low to exclude active disease reliably.

### 4.2. Role of Biomarkers

Combined marker panels outperformed CRP alone in one cohort [18], which illustrates the limits of relying on traditional acute-phase reactants with high sensitivity (about 85%) but modest specificity (about 61%) [7]. IL-8 correlates with disease severity and mucosal IL-8 with relapse [17,19], but correlation is not sufficient to call it the most reliable cytokine biomarker. Its value relative to fecal calprotectin, particularly within GIUS-based monitoring, must be tested prospectively.

### 4.3. Synergy of Combined GIUS and Biomarker Assessment

GIUS and biomarkers have complementary limitations: GIUS gives localized, transmural anatomical information, whereas biomarkers give quantitative, systemic or luminal inflammatory readouts that may help in detecting subclinical disease and predicting relapse [25,26,33]. Composite ultrasound scores likewise appear more informative than single parameters [9,10]. The combination of BWT and calprotectin is the most frequently studied integrated approach, but its supporting data come from small studies and its NPV is modest.

### 4.4. Clinical Implications and Positioning

Guidelines (ACG 2025, AGA 2024) recognize intestinal ultrasound as a valuable tool for measuring disease activity and monitoring treatment response [1,3]. The current evidence supports GIUS as a complementary tool alongside endoscopy in selected situations:

- Serial monitoring of established disease in clinical remission, where GIUS with biomarkers may help identify patients who need endoscopic evaluation [28,36].
- Pre-screening for clinical trials, where segmental BWT thresholds may reduce screen failures [37].
- Rapid assessment of treatment response, particularly in inpatient settings or during dose optimization, with BWT and Doppler changes detectable within days to weeks [29].
- Small-bowel disease, where GIUS approaches MRE in sensitivity for terminal ileal involvement and offers point-of-care availability [32].

**Figure 4:**
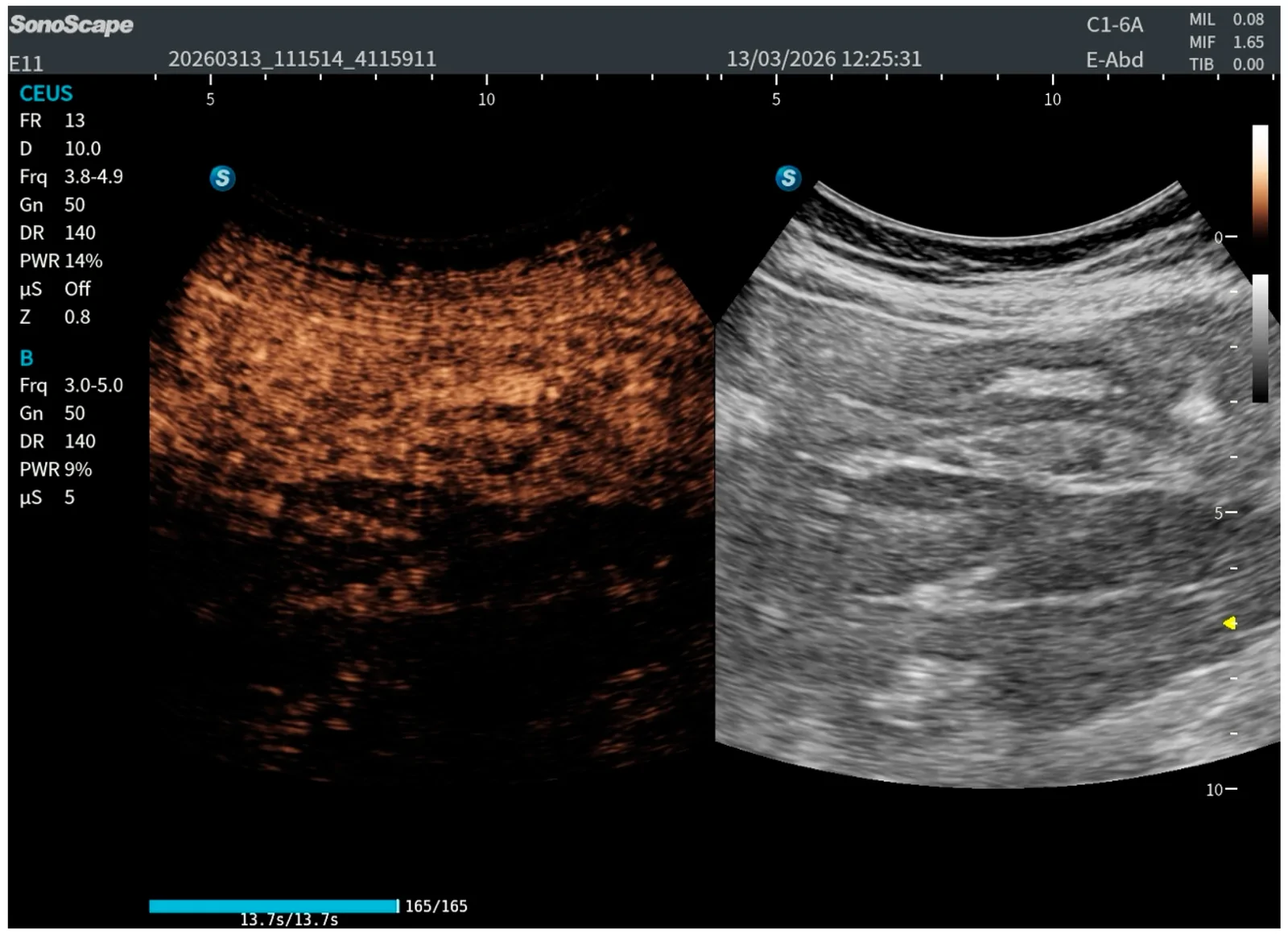
CEUS of the colon in a patient with suspected IBD flare (dual-panel display: contrast-enhancement map on the **left** and B-mode image on the **right**). Early post-contrast phase (approx. 13.7 s) demonstrates diffuse hyperenhancement of the colonic wall consistent with inflammatory hyperperfusion. The corresponding B-mode image shows bowel wall thickening with features of edema.

Because comparative outcome studies are missing and the reported NPVs are modest, the data do not support GIUS as a first-line replacement for endoscopy or the safe deferral of endoscopy. Endoscopy should remain the reference standard when results are discordant or when management depends on mucosal status, given the potentially irreversible consequences of missed active disease (bowel damage, strictures, resection). Figure 4 shows an example view of this.

### 4.5. Quality of Evidence and Knowledge Gaps

1. No randomized comparisons of monitoring strategies. We found no randomized trials comparing GIUS–biomarker monitoring with endoscopy-based monitoring for long-term outcomes (hospitalization, surgery, disability, quality of life) [32,35]. The AGA guidelines identify insufficient evidence for biomarker-based monitoring instead of endoscopy as a key gap [15]. Pragmatic trials in patients in clinical remission are needed.
2. Optimal biomarker combinations and thresholds. Fecal calprotectin < 250 µg/g correlates with endoscopic remission [15,16], but the incremental value of adding cytokine panels (IL-8, IL-10, IFN-γ) to GIUS plus calprotectin is undefined. Validation should show whether multiplex measurement justifies its cost and complexity, especially for predicting relapse after endoscopic remission.
3. Standardization and training. Ultrasound protocols, scoring systems (IBUS-SAS, BUSS, Simple-US, SUS-CD) and operator training vary between studies, limiting comparability [23,31]. Consensus on scanning technique, transducer frequency (typically 7–12 MHz) and scoring is needed, along with structured training and competency assessment, because ultrasound is operator-dependent.
4. Mild endoscopic disease and the rectum. GIUS performs less well in mild disease and in the rectum [6]. Supplementary parameters or composite indices are needed for these situations, in which clinical decisions are often hardest.
5. Transmural healing as a target. Cross-sectional imaging findings beyond the mucosa may predict outcomes better than endoscopic mucosal assessment alone [38]. Unlike endoscopy, GIUS can assess transmural inflammation, mesenteric changes and complications such as strictures, fistulas and abscesses [3,38]. Prospective studies should test whether GIUS-defined transmural healing predicts long-term outcomes better than mucosal healing.
6. Cost-effectiveness and implementation. CEUS is limited by contrast-agent availability, regulatory differences between countries and cost [4,32]. Formal cost-effectiveness analyses of GIUS-based versus endoscopy-based strategies, including equipment, training, procedure time and downstream utilization, are lacking [35].
7. Advanced biomarkers and artificial intelligence. The synergy between GIUS, advanced biomarkers (cytokines, metabolomics, microbiome signatures) and AI-assisted interpretation is unexplored. Machine-learning algorithms integrating GIUS parameters, biomarkers and clinical variables might improve prediction and reduce operator dependence, but none has been validated in the studies mapped here.

### 4.6. Limitations

We did not perform a formal risk-of-bias assessment, so the reliability of individual findings is unknown. Included studies were heterogeneous in populations, disease phenotypes, ultrasound protocols and outcome definitions, which limits direct comparison and any meta-analytic pooling. Most studies examined moderate-to-severe disease, with limited data on mild activity. Ultrasound is operator-dependent, and although reported interobserver agreement is encouraging, it depends on training. Long-term outcome data comparing GIUS-guided with endoscopy-guided management are lacking, preventing conclusions about clinical equivalence. The search was restricted to English-language publications from 2018, and the mandatory biomarker block may have missed studies comparing GIUS with endoscopy only. Publication bias may favor positive correlations. Sensitivity and specificity ranges come from published meta-analyses and individual studies and were not pooled by us.

The search strategy required a biomarker term in every query, so studies that compared GIUS with endoscopy alone and did not mention biomarkers in their titles, abstracts or indexing terms may have been missed. The 15 included studies therefore should not be read as a complete set of primary data on GIUS accuracy against endoscopy, particularly for B-mode and Doppler parameters. For this reason, the sensitivity and specificity ranges reported here come from published meta-analyses with their own search strategies [4,6,7], and a search without the biomarker block would be needed to map GIUS-versus-endoscopy accuracy comprehensively.

### 4.7. Implications for Future Research

Future work should prioritize: (1) pragmatic randomized trials with 2–3 years of follow-up comparing GIUS–biomarker with endoscopy-based monitoring in patients in clinical remission, powered for hospitalization, surgery and disability; (2) prospective validation cohorts evaluating the incremental value of multiplex cytokine panels beyond GIUS plus calprotectin for predicting relapse; (3) multicenter initiatives establishing consensus protocols, training curricula and competency frameworks; (4) longitudinal studies correlating GIUS-defined transmural healing with long-term course independent of endoscopic findings; (5) cost-effectiveness analyses from health-system and societal perspectives; and (6) development and validation of AI tools for automated GIUS interpretation and multimodal data integration. Combined GIUS–biomarker assessment may prove a viable complementary option in selected scenarios, but definitive evidence from randomized trials is needed before any move away from endoscopy-based monitoring.

## 5. Conclusions

In this scoping review, GIUS showed promising diagnostic performance for IBD activity against endoscopy, with published meta-analytic ranges of 85–91% sensitivity and 83–96% specificity. BWT > 3 mm was the most frequently evaluated parameter, while Limberg grading and quantitative CEUS parameters (TTP, PI) added complementary but less standardized information on severity. Combining GIUS with biomarkers, particularly calprotectin, achieved accuracies of about 88–92% in small studies and may help predict treatment response and relapse, but IL-8 and other cytokines remain hypothesis-generating. Given the heterogeneity, the absence of quality appraisal, the modest NPVs and the lack of randomized comparisons, GIUS should be regarded as a complementary tool alongside endoscopy, not a replacement, and current data do not justify deferring endoscopy. Standardized protocols, validated scores and randomized trials of monitoring strategies are needed. Future research should also validate multiplex cytokine panels (IL-8, IL-10, IFN-γ), develop AI-assisted interpretation, and investigate transmural healing as a therapeutic target.

## Figures and Tables

**Table 1:** Key included studies contributing to the quantitative findings reported in Section 3. SES-CD, Simple Endoscopic Score for Crohn’s Disease; FC, fecal calprotectin; TTP, time to peak; Acc, accuracy; Sens, sensitivity; Spec, specificity.

#	First Author, Year	Design	Population (IBD Type, *n*)	GIUS Modality	Comparator	Cut-Off/Score	Key Findings
1	de Voogd 2022 [8]	Prospective longitudinal cohort	UC, moderate–severe, on tofacitinib (*n* = 30)	B-mode (BWT)	Endoscopic Mayo score	2.8 mm; 3.9 mm	ρ = 0.68; AUC 0.87 (remission), 0.92 (improvement)
2	Liu 2020 [30]	Cross-sectional	CD (*n* = 125)	B-mode + Limberg	SES-CD	Limberg grade	ρ = 0.709
3	Ripollés 2021 [9]	Multicenter	CD (*n* = 72)	B-mode + Doppler + CEUS score	Endoscopy	Composite score	AUC 0.972 (with CEUS), 0.923 (without)
4	Servais 2022 [11]	Prospective	CD (*n* = 17)	B-mode, CEUS	Surgical histopathology; MRE	TTP, rise time	AUC 0.88, 0.86
5	Marin 2021 [10]	Prospective	IBD (*n* = 52)	Composite score (BWT, Limberg, extent, elastography)	Endoscopy	≥7 points	Sens 86.85%, spec 94%
6	Parihar 2025 [25]	Cross-sectional	CD (*n* = 45)	B-mode	SES-CD; calprotectin	BWT > 3 mm + FC > 250 µg/g	Ileum 88.6/78.9%; descending colon 80.9/87.7%
7	Goodsall 2024 [26]	Cohort	UC (*n* = 56)	IUS + calprotectin	Histology	Composite	Acc 88%; NPV 57%
8	Patel 2026 [27]	Cohort	Pediatric IBD (*n* = 92)	IUS + clinical	Biochemical activity	—	AUC CD 0.71→0.90; UC 0.83→0.92
9	Allocca 2022 [33]	Prospective, 12 months	CD (*n* = 225)	Bowel ultrasound score	Clinical outcomes	>3.52	OR 6.97 (2.87–16.93)
10	Dragoni 2023 [31]	Prospective exploratory	CD (*n* = 60)	Several US scores	Endoscopy	IBUS-SAS, etc.	IBUS-SAS ρ = 0.87

**Table 2:** Meta-analyses and systematic reviews used as sources of pooled or summary estimates.

Source	Type	Estimate(s) Attributed to It
Sagami 2021 [6]	Systematic review/meta-analysis	BWT > 3 mm colorectal sens 86.4%, spec 88.3%; rectum 74.5%/69.5%
Huynh 2024 [7]	Systematic review/meta-analysis	CRP sens ~85%, spec ~61%; IUS vs. endoscopy
Bharadwaj 2026 [4]	Systematic review/meta-analysis	GIUS/CEUS accuracy; DOR 75.52 for CEUS
Josefsen 2025 [29]	Systematic review	BWT changes and outcomes in UC
Samnani 2025 [34]	Systematic review/meta-analysis	Postoperative CD recurrence
Innocenti 2025 [23]	Systematic review	Validation of IUS scores

## Data Availability

All data analyzed are presented in this article and its Supplementary Materials.

## Supplementary Materials

The following supporting information can be downloaded at https://www.mdpi.com/article/10.3390/jcm15207984/s1, Table S1. Preferred Reporting Items for Systematic reviews and Meta-Analyses extension for Scoping Reviews (PRISMA-ScR) Checklist.

## Appendix A. Search Strings

The core query in Section 2.2 was formulated for each database as follows. Limits in all databases: English language, 1 January 2018–31 March 2026.

- **PubMed:** (“Inflammatory Bowel Diseases”[Mesh] OR “Crohn Disease”[Mesh] OR “Colitis, Ulcerative”[Mesh] OR “inflammatory bowel disease”[tiab] OR “Crohn’s disease”[tiab] OR “ulcerative colitis”[tiab]) AND (“Ultrasonography”[Mesh] OR “intestinal ultrasound”[tiab] OR “gastrointestinal ultrasound”[tiab] OR GIUS[tiab] OR “bowel ultrasound”[tiab] OR “contrast-enhanced ultrasound”[tiab] OR CEUS[tiab] OR “Doppler ultrasound”[tiab]) AND (“disease activity”[tiab] OR endoscopy[tiab] OR “endoscopic activity”[tiab]) AND (biomarkers[tiab] OR “C-reactive protein”[tiab] OR “fecal calprotectin”[tiab] OR cytokines[tiab] OR “IL-8”[tiab] OR “IL-6”[tiab] OR “IL-10”[tiab])
- **Scopus:** TITLE-ABS-KEY of the same four concept blocks, combined with AND.
- **Web of Science Core Collection:** TS = the same four concept blocks, combined with AND.
- **Embase:** the same four blocks using Emtree terms (exploded) plus title/abstract/keyword free-text terms, combined with AND.
